# From an Empty Stomach to Anxiolysis: Molecular and Behavioral Assessment of Sex Differences in the Ghrelin Axis of Rats

**DOI:** 10.3389/fendo.2022.901669

**Published:** 2022-06-16

**Authors:** Stina Börchers, Jean-Philippe Krieger, Ivana Maric, Jil Carl, Maral Abraham, Francesco Longo, Mohammed Asker, Jennifer E. Richard, Karolina P. Skibicka

**Affiliations:** ^1^Department of Physiology, Institute for Neuroscience and Physiology, University of Gothenburg, Gothenburg, Sweden; ^2^Wallenberg Centre for Molecular and Translational Medicine, University of Gothenburg, Gothenburg, Sweden; ^3^Department of Nutritional Sciences, Pennsylvania State University, University Park, PA, United States

**Keywords:** ghrelin, anxiety-like behavior, sex difference, GHSR, LEAP-2, JMV2959, acoustic startle, fasting

## Abstract

Ghrelin, a stomach-produced hormone, is well-recognized for its role in promoting feeding, controlling energy homeostasis, and glucoregulation. Ghrelin’s function to ensure survival extends beyond that: its release parallels that of corticosterone, and ghrelin administration and fasting have an anxiolytic and antidepressant effect. This clearly suggests a role in stress and anxiety. However, most studies of ghrelin’s effects on anxiety have been conducted exclusively on male rodents. Here, we hypothesize that female rats are wired for higher ghrelin sensitivity compared to males. To test this, we systematically compared components of the ghrelin axis between male and female Sprague Dawley rats. Next, we evaluated whether anxiety-like behavior and feeding response to endogenous or exogenous ghrelin are sex divergent. In line with our hypothesis, we show that female rats have higher serum levels of ghrelin and lower levels of the endogenous antagonist LEAP-2, compared to males. Furthermore, circulating ghrelin levels were partly dependent on estradiol; ovariectomy drastically reduced circulating ghrelin levels, which were partly restored by estradiol replacement. In contrast, orchiectomy did not affect circulating plasma ghrelin. Additionally, females expressed higher levels of the endogenous ghrelin receptor GHSR_1A_ in brain areas involved in feeding and anxiety: the lateral hypothalamus, hippocampus, and amygdala. Moreover, overnight fasting increased GHSR_1A_ expression in the amygdala of females, but not males. To evaluate the behavioral consequences of these molecular differences, male and female rats were tested in the elevated plus maze (EPM), open field (OF), and acoustic startle response (ASR) after three complementary ghrelin manipulations: increased endogenous ghrelin levels through overnight fasting, systemic administration of ghrelin, or blockade of fasting-induced ghrelin signaling with a GHSR_1A_ antagonist. Here, females exhibited a stronger anxiolytic response to fasting and ghrelin in the ASR, in line with our findings of sex differences in the ghrelin axis. Most importantly, after GHSR_1A_ antagonist treatment, females but not males displayed an anxiogenic response in the ASR, and a more pronounced anxiogenesis in the EPM and OF compared to males. Collectively, female rats are wired for higher sensitivity to fasting-induced anxiolytic ghrelin signaling. Further, the sex differences in the ghrelin axis are modulated, at least partly, by gonadal steroids, specifically estradiol. Overall, ghrelin plays a more prominent role in the regulation of anxiety-like behavior of female rats.

## Introduction

Ghrelin, a stomach-derived hormone, has been recently identified as a potential mediator of stress and anxiety disorders (1, 2). It is already well-recognized for its role in promoting feeding and glucoregulation by acting on the growth-hormone secretagogue receptor 1A (GHSR_1A_) (3–5). Circulating levels of ghrelin rise during acute or chronic negative energy balance induced by fasting or caloric restriction, and before expected meals to increase food intake (4, 6–9). Moreover, the recent discovery of an endogenous antagonist for GHSR_1A_, the liver-expressed antimicrobial peptide 2 (LEAP-2) added an important new nuance to the endogenous regulation of ghrelin singling. LEAP-2 inversely correlates with circulating ghrelin (4, 10, 11). Ghrelin, LEAP-2, and GHSR_1A,_ along with the enzyme activating ghrelin, GOAT, form key elements of the ghrelin axis.

Gut peptides influence emotional reactivity and mood, in addition to their presumed primary role in control of feeding behavior. This is certainly the case for ghrelin. Several findings indicate that the function of ghrelin extends to the control of stress and anxiety. Ghrelin levels are associated with corticosterone concentration (12, 13). Further, the expression of GHSR_1A_ in brain areas involved in anxiety, such as the amygdala (14, 15), together with anxiolytic and antidepressant effects (1) of calorie restriction and ghrelin administration clearly suggest a function beyond appetite regulation. Furthermore, recombinant adeno-associated-virus-mediated GHSR_1A_ overexpression in the basolateral amygdala reduces anxiety-like behavior in the common rodent models of anxiety-like behavior, the open field (OF) and the elevated plus maze (EPM). It does so through the receptor’s high constitutive activity (2). In addition, to the acute effects of ghrelin administration or caloric restriction, chronic exposure to psychosocial stress upregulates circulating ghrelin in rats for up to 7 months after exposure, and in humans at least for 4.5 years (16). On the other hand, obese humans and those suffering from depression, both conditions highly comorbid with anxiety, present with reduced circulating levels of ghrelin (17–19). Similarly, rodents lacking the ghrelin receptor display more severe symptoms of depression when challenged with stress (1). Likewise, a polymorphism in the ghrelin gene that decreases circulating ghrelin increases the risk for developing major depressive disorder (18). Therefore, alterations in ghrelin signaling contribute both to disease development as well as response.

To date, most studies of ghrelin’s effects on anxiety-like behavior in rodents have been conducted almost exclusively on males. Thus, and the role of ghrelin in female anxiety-like behavior is yet to be unraveled. However, several studies documented sex differences in orexigenic effects of ghrelin or ghrelin receptor manipulations: Although all indicated clear sex differences, one found ghrelin’s effect on feeding to be more potent in females (20), while the other reported an attenuated effect of ghrelin in females compared to males (21). In the latter study, a higher sensitivity to ghrelin’s orexigenic effects was reported in intact males and ovariectomized rats compared to intact females when administered systemically and centrally (21). Conversely, direct delivery of ghrelin to the lateral hypothalamus (LH) increases food intake and motivation for sucrose in both males and females, but increases food seeking behavior only in females (22). These behavioral differences in feeding effects of ghrelin are further supported by higher expression of GHSR_1A_ in the LH of females. Additionally, females seem to be more dependent on endogenous ghrelin since blockade of GHSR_1A_ in the LH results in reduced food intake and motivated behavior only in females, but not males. In female mice, ghrelin treatment induced a stronger electrical stimulation of fibers from the nucleus of the solitary tract to the arcuate nucleus (ARC) than in males (20). Given this potential disparity of ghrelin’s role in the control of feeding between sexes, it is likely that ghrelin exerts sex divergent effects in other behaviors as well.

The goal of the current study is to elucidate potential sex differences in molecular and behavioral aspects of ghrelin that could drive sex differential susceptibility to anxiety and eating disorders. To achieve this, we first analyzed different molecular components of the ghrelin axis, including circulating ghrelin, GHSR_1A_ expression in brain areas involved in anxiety and feeding, and hepatic expression of ghrelin’s endogenous antagonist LEAP-2, in male and female rats. Furthermore, we measured circulating ghrelin levels and gastric expression of ghrelin in intact and ovariectomized females to explore the role of estradiol with the help of estradiol replacement. Behavioral comparison of sex differences in ghrelin responses was conducted after overnight fasting increased circulating ghrelin, or exogenous ghrelin application, and anxiety-like behavior was assessed with the EPM, OF, and acoustic startle response (ASR) test. Finally, to evaluate sex differences in response to endogenous ghrelin signaling, anxiety-like behavior was measured after acute pharmacological blockade of GHSR_1A_ in fasted rats.

## Material and Methods

### Animals

Age-matched female and male Sprague-Dawley rats (8 weeks of age at arrival, Charles River, Italy) were pair-housed at 21-22°C and 55-65% humidity under a 12-hour light/dark cycle (lights on at 7:00 AM) with water and chow (Envigo, 2018 Teklad Global) available *ad libitum*, unless stated otherwise. Males and females were housed in different rooms. To reduce stress, all rats were handled frequently. All animal procedures were carried out with ethical permission from the Animal Welfare Committee of the University of Gothenburg and Jordbruksverket, in accordance with legal requirements of the European Community (Decree 35-970/96, 137/15, and 1-2019). All efforts were made to minimize suffering.

### Drugs and Caloric Restriction

Overnight fasting was performed to increase endogenous levels of ghrelin. Therefore, food was removed from cages at 6pm the day before the experiment. Experiments started at 9am. Controls were fed *ad libitum*.

Acyl-Ghrelin was purchased from Bionordika (Solna, Sweden), solved in saline (0.7%), and administered intraperitoneally (IP) at a dose of 0.33mg/kg in *ad libitum* fed rats.

JMV2959 hydrochloride is a GHSR_1A_ antagonist (23) and was purchased from MedChemtronica AB (Sollentuna, Sweden). It was dissolved in saline (0.7%), and administered IP at a dose of 3mg/kg in overnight fasted rats.

For each drug, a different cohort of age-matched rats was used to avoid potential habituation to the anxiety-tests. The number of subjects was always 12 per group. The response to the drugs was always tested within subject per sex, and drugs were administered in a counterbalanced order with at least 2 days separating each injection day. The injection volume for all drugs was 1ml/kg. Controls received vehicle only. Rats were habituated to IP injections prior to the respective experiments.

### Food Intake Measurement

Immediately after drug administration (ghrelin or JMV2959) or overnight fasting, rats were given access to pre-weighed chow. Total food intake was measured 1 hour after access to determine whether IP administration of ghrelin results in sex divergent responses in rats and also to validate that the dose used in the anxiety-tests has an effect on feeding.

### Assessment of Anxiety-Like Behavior

To assess ghrelin’s effects on anxiety-like behavior, rats were tested in the OF, ASR and/or EPM following drug treatment (ghrelin or JMV2959) or caloric restriction as described above. Rats had a 1-week break after each anxiety test to avoid a potential effect of stress.

#### Elevated Plus Maze

Anxiety-like behavior was evaluated in an EPM comprised of two open and two closed arms (45x10cm) and a platform in the middle (10x10cm), elevated 50cm above the ground. The rat was placed in the junction and allowed to move freely for five minutes, which was recorded using a camera mounted above the maze. The EPM was lit with a light intensity of 35 lux. Movement was immediately tracked and prepared for further analysis using EthoVision 13 XT. Data were evaluated for time spent in the open arm of the maze and distance travelled. This test is based on the innate aversion of rats against bright and open spaces, and a rat is considered more anxious when it spends less time in the open arms of the apparatus.

#### Open Field

As an additional measurement of anxiety-like behavior, rats were placed in a brightly lit OF (100x100x40cm) and allowed to freely explore for 15 minutes. Levels of anxiety were assessed by the amount of time spent and locomotor activity in the central part of the arena. The OF was lit with a light intensity of 35 lux. Movement was recorded with a camera mounted above the arena and tracked with EthoVision XT, data were evaluated for time spent in the central area of the OF and distance travelled. This test, like EPM, is also based on the innate aversion of rats against bright and open spaces. A rat is considered more anxious when it spends less time in the open area of the apparatus.

#### Acoustic Startle Response

To assess the ASR, an adaptation of the protocol by Maniscalco and colleagues (24) was applied using the SR Lab Startle Response system (San Diego Instruments, San Diego, USA). Rats were placed into a Plexiglas cylinder (Ø13 cm for rats > 450 g and Ø 9 cm for rats < 450 g). Each run started with 5 minutes of habituation, followed by three acoustic stimulus intensities (90, 95, and 105dB), presented 10 times in a randomized order with inter stimulus intervals between 20s and 40s. The ASR to each stimulus was transduced by an accelerometer and recorded as 100 1ms readings, beginning at the onset of each startle stimulus. Average peak startle amplitude was evaluated for each stimulus intensity without illumination. Higher startle amplitude is considered to indicate increased anxiety-like behavior.

### Gonadectomies

Gonadectomies were performed on male (n = 20) and female (n = 49) rats at 10 weeks of age. Surgical anesthesia was achieved by IP administration of ketamine (18.75 mg/kg) and xylazine (2.5 mg/kg). Males were bilaterally orchiectomized (ORX) by ablating the testes from the surrounding epididymis using a ligature around the blood vasculature and efferent ductules. Females were bilaterally ovariectomized (OVX) by a ligature around the blood vasculature and fallopian tube. Uterine tubes and adipose tissue were returned into the abdominal cavity. The intact male and female control groups received anesthesia only. After surgeries, rats were injected subcutaneously with an analgesic (Metacam, 2mg/kg).

### Estradiol Replacement

To assess the effect of estradiol on endogenous ghrelin levels, a cohort of females was divided into intact (n = 12) and OVX female groups (n = 24). Half of the OVX animals received estradiol replacement. Replacement began 4 days after surgeries. 17β-Estradiol-3-benzoate was purchased from Sigma Aldrich, dissolved in sesame oil, and administered subcutaneously at a dose of 2μg/100μl/animal every 4th day over a period of three months to mimic the natural fluctuations of the estrous cycle. Intact females and OVX controls received vehicle only. The estradiol replacement protocol was based on the work of Asarian and colleagues (25), where it was previously shown to successfully restore the body weight of OVX rats to the level of intact animals.

### Tissue Collection and Analysis

For tissue collection, rats were lightly anesthetized with isoflurane (Baxter AB, Sweden), and decapitated at 30 weeks of age. Trunk blood was immediately collected and mixed with AEBSF protease inhibitor (1mg/mL) (Thermo Fisher, Sweden). Blood was left to clot at room temperature for 30 minutes followed by centrifugation at 3000 x g and 4°C for 15 minutes to obtain serum. The latter was acidified with 1M HCl to a final concentration of 0.05N. Blood samples with hemolysis were discarded. Additionally, blood glucose levels were determined in fasted vs. *ad libitum* fed rats using a drop of tail vein blood and a glucose meter before sacrifice (Bayer, Germany). Brains were rapidly collected and flash frozen in dry-ice cooled isopentane, liver and the stomach corpus were dissected and frozen in liquid nitrogen. All collected samples were stored at -80°C until further processing.

Brains were sectioned in 60µm sections using a cryostat (Leica 3050S; Leica Biosystems, Germany), and hippocampus, amygdala, bed nucleus of stria terminalis (BNST), paraventricular nucleus (PVN), LH, dorso-vagal complex (DVC), and arcuate nucleus (ARC) were dissected bilaterally using disposable biopsy punches with plungers (INTEGRA, USA).

Acylated ghrelin in serum was measured using a rat ghrelin (active) ELISA kit according to manufacturer’s instructions (Millipore, Billerica, USA). Total RNA from brain samples was extracted using RNeasy Lipid Tissue Mini Kit (Qiagen). For other samples Trizol (Invitrogen) extraction was applied. RNA quality and quantity were assessed using a Nanodrop 1000 (NanoDrop technologies) prior to cDNA synthesis, which was performed using the iScript cDNA Synthesis kit (Bio-Rad). Gene expression levels of GHSR_1A_ (Rn00821417_m1), ghrelin (Rn00572319_m1), and LEAP-2 (Rn03648192_m1) were quantified through Taqman RT-qPCR, with beta actin (Rn00667869_m1) as a reference gene for brain samples, GAPDH (Rn01775763_g1**)** for liver samples, and HPRT1 (Rn01527840_m1) for stomach samples. Gene expression values were calculated based on the ΔΔCt method (26).

### Statistical Analysis

All data are presented as mean ± standard error of the mean (SEM). Means were compared with Student’s t-test for comparisons of two groups, one- or two-way analysis of variance (ANOVA) with *post-hoc* Holm-Sidak tests when appropriate (GraphPad Prism 8 Software, Inc, USA). One-way ANOVA was used for comparison of two or more treatment groups of the same sex (one independent variable, e.g. treatment), two-way ANOVA for the comparison of the effect of two independent variables (e.g. sex and treatment). The latter was performed as a repeated measures analysis for food intake and behavioral experiments. If a significant main factor effect or interaction of sex and treatment was detected, ANOVA was followed up with the *post-hoc* Holm-Sidak test to compare groups. To control for total locomotor activity in the EPM and OF, an analysis of covariance (ANCOVA) was performed using the *car* package for R (v. 4.3.1). *p*-values lower than 0.05 were considered statistically significant. *, **, ***, and **** refer to *p*-values smaller than 0.05, 0.01, 0.001, and 0.0001 respectively.

## Results

### Molecular Components of Ghrelin Axis Are Sexually Dimorphic and Regulated by Ovarian Hormones

**Figure 1:**
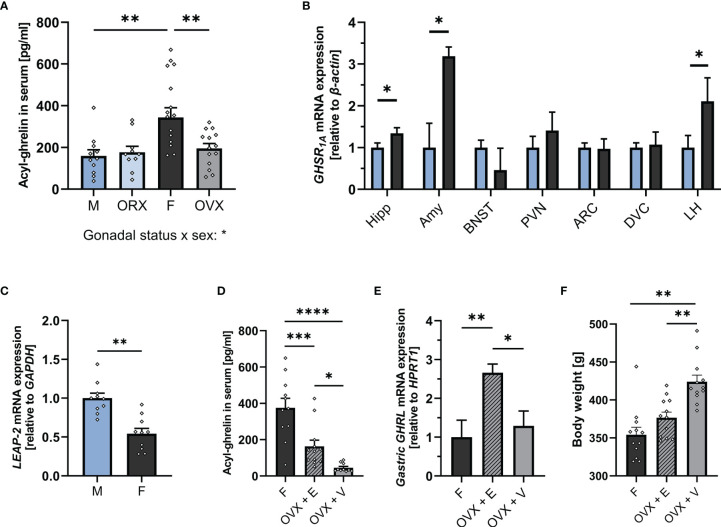
The ghrelin axis at baseline is sexually dimorphic. Intact females (n = 17) had significantly higher levels of acyl-ghrelin in serum compared to males (n = 11) and OVX females (n = 14). Ghrelin levels in ORX males (n = 11) did not differ from intact males **(A)**. Females (n = 12) had higher GHSR_1A_ mRNA expression in the Hipp, Amy, and LH compared to males (n = 12) **(B)**. Males (n = 10) expressed significantly more hepatic LEAP-2 compared to females (n = 10) **(C)**. OVX females had lower serum ghrelin levels compared to intact females. Estradiol replacement was able to partially restore ghrelin levels (n = 11 per group) **(D)**. OVX females which received estradiol replacement had a higher expression of ghrelin in the stomach compared to intact females and OVX females with vehicle treatment (n = 11 per group) **(E)**. OVX rats that received vehicle only, had a significantly higher body weight on the day of tissue collection compared to OVX rats with estradiol replacement and intact females (n = 12 per group) **(F)**. All data are presented as mean ± SEM. *p < 0.05, **p < 0.01, ***p < 0.001, ****p < 0.0001 compared with respective controls. ORX, orchiectomized; OVX, ovariectomized; GHSR_1A_, ghrelin receptor; Hipp, Hippocampus; Amy, Amygdala; BNST, Bed nucleus of stria terminalis; PVN, paraventricular nucleus; ARC, arcuate nucleus; DVC, dorso-vagal complex; LH, lateral hypothalamus; LEAP-2, liver-expressed antimicrobial peptide 2; GAPDH, glyceraldehyde-3-phosphate dehydrogenase (housekeeping gene); OVX + V, ovariectomized females with vehicle treatment; OVX + E, ovariectomized females with estradiol replacement. GHRL, ghrelin (gene).

To determine whether there are potential sex differences in key molecular components of the ghrelin axis, we measured acyl-ghrelin levels in blood serum, hepatic expression of the endogenous antagonist LEAP-2, and GHSR_1A_ expression in brain areas involved in anxiety and feeding in *ad libitum* fed male and female rats. Females had nearly two-fold higher plasma ghrelin levels compared to males (**Figure 1A**), and had lower expression of the newly discovered endogenous ghrelin receptor antagonist, LEAP-2, in the liver (**Figure 1C**).In the brain, GHSR_1A_ mRNA expression was significantly higher in the hippocampus, amygdala, and LH of female rats. On the other hand, GHSR_1A_ expression was similar between males and females in the BNST, PVN, ARC and DVC (**Figure 1B**).

To understand whether the substantial difference in plasma ghrelin levels between the sexes is mediated by gonadal hormones, ghrelin levels were measured in OVX females and ORX males. OVX resulted in markedly lower plasma ghrelin levels in females, indistinguishable from those found in males (**Figure 1A**). In contrast, ORX did not affect ghrelin levels in males (**Figure 1A**). Ovariectomy removes all gonadal hormones, and thus does not allow conclusions on specific contribution of individual ovarian hormones. Here, we hypothesized that the increased plasma ghrelin levels found in females are specifically mediated by estradiol. In order to evaluate this hypothesis, plasma levels of ghrelin were measured in a new cohort of female rats with OVX or OVX with estradiol replacement. Results partly support our hypothesis as OVX rats receiving estradiol replacement had four-fold higher ghrelin levels compared to OVX vehicle rats (**Figure 1D**), clearly indicating that estradiol is sufficient to increase circulating ghrelin levels. However, compared to intact females, OVX rats with estradiol replacement still had substantially lower ghrelin levels. Additionally, results from this second cohort confirm our original finding (**Figure 1A**) that OVX markedly suppresses circulating ghrelin. To further determine the mechanism of estradiol-mediated ghrelin elevation, ghrelin gene expression was measured in the stomach of intact females, OVX females, and OVX females with estradiol replacement. Surprisingly, OVX did not alter ghrelin mRNA levels in rats that received vehicle only, but estradiol replacement led to a 2.5-fold increase when compared to intact females (**Figure 1E**). Additionally, OVX rats that received vehicle only, had a significantly higher body weight on the day of tissue collection compared to OVX rats with estradiol replacement and intact females (**Figure 1F**). However, no significant relationship of body weight and ghrelin levels was detected by linear regression, and the difference in circulating ghrelin levels remained when only comparing rats of a similar body weight (**Supplementary Figures 1A**, **B** respectively).

### Sex Divergent Effects of Overnight Fasting on the Molecular Aspects of Ghrelin Axis

**Figure 2:**
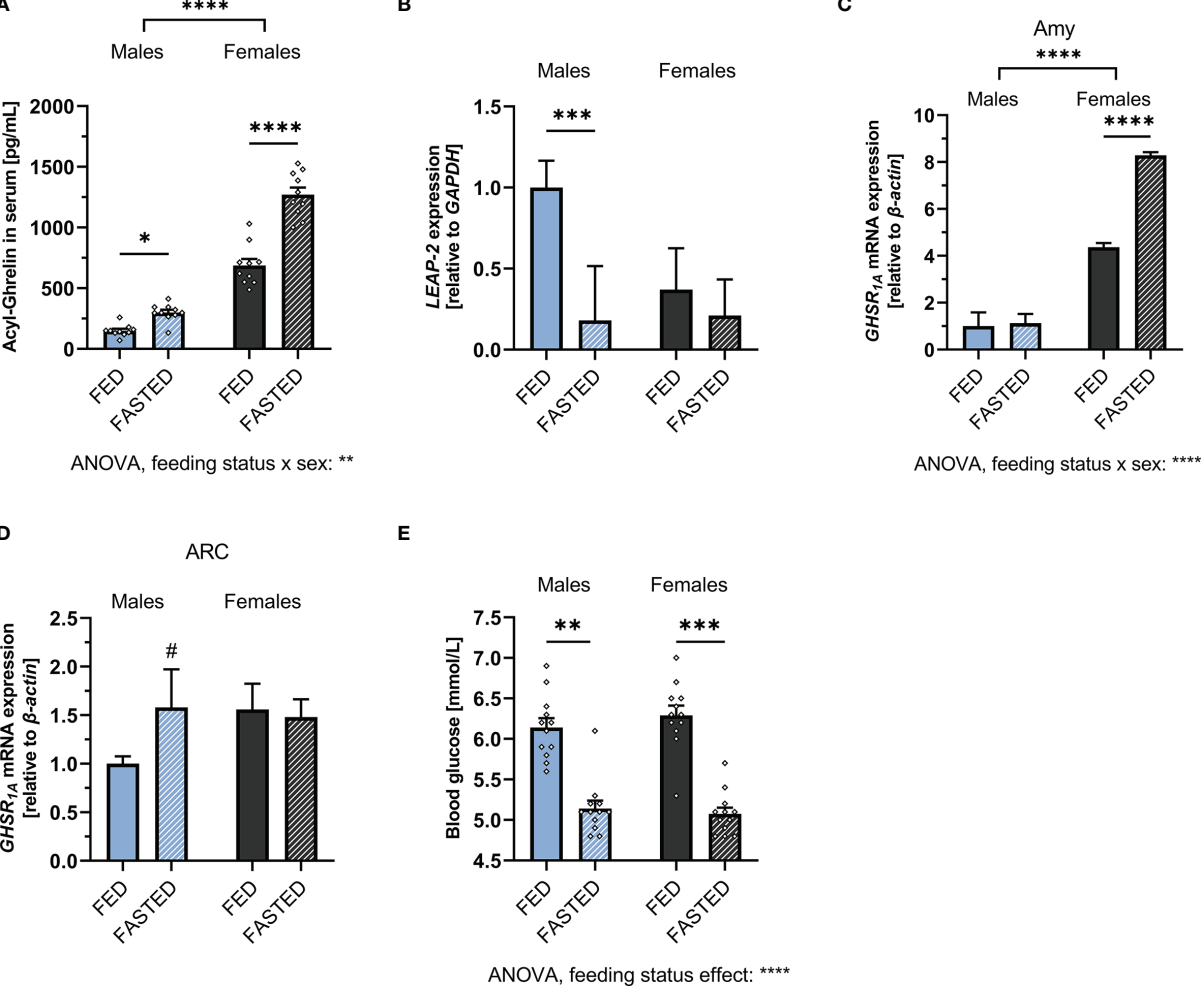
Alterations of the ghrelin axis induced by overnight fasting. Fasting increased circulating acyl-ghrelin levels in both males and females (n = 10 per group) **(A)**. Hepatic LEAP-2 mRNA expression was significantly decreased after fasting in males, but not females (n = 10 per group) **(B)**. GHSR_1A_ mRNA expression was increased in the amygdala of fasted females, but not males (n = 10 per group). **(C)**. A trend for upregulation of GHSR_1A_ expression in the ARC was detected in fasted males, but not females (n = 12 per group) **(D)**. Overnight fasting decreased blood glucose in both males and females (n = 12 for each group) **(E)**. All data are presented as mean ± SEM. ^#^*p* < 0.1, *p < 0.05, **p < 0.01, ***p < 0.001, ****p < 0.0001 compared with respective controls. GHSR_1A_, ghrelin receptor; LEAP-2, liver-expressed antimicrobial peptide 2; GAPDH, glyceraldehyde-3-phosphate dehydrogenase (housekeeping gene); Amy, Amygdala; ARC, arcuate nucleus.

When challenged by overnight fasting, both males and females responded with a significant increase in ghrelin levels (**Figure 2A**). However, both fed and fasted plasma ghrelin levels were still significantly lower in males compared to females, with females having five-fold higher ghrelin levels while fasted compared to males. Two-way ANOVA revealed a strong effect of sex, feeding status, as well as a sex x feeding status interaction (see the **Supplementary Table 1** for detailed ANOVA results). Hepatic LEAP-2 expression was significantly downregulated in fasted males but not females (**Figure 2B**). GHSR_1A_ expression was upregulated in the amygdala of fasted females only (**Figure 2C**), whereas a trend for an upregulation was found in the ARC of fasted males only (**Figure 2D**).

To ensure that both sexes were equally metabolically challenged by the fasting regimen, blood glucose was measured during fed and fasted state. Blood glucose was decreased by fasting to a similar extent in both males and females (**Figure 2E**). Two-way ANOVA revealed a significant overall effect of feeding status on blood glucose levels (**Supplementary Table 1** for details).

### Sex Differences in Food Intake After Enhancing or Inhibiting Ghrelin Signaling

**Figure 3:**
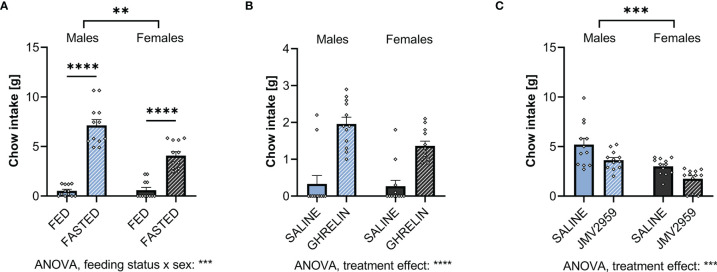
Ghrelin and overnight fasting increases and blockage of ghrelin signaling decreases food intake in both sexes. When fasting male and female rats overnight, both sexes consume more chow within 1h when compared to *ad libitum* fed controls **(A)**. Treating *ad libitum* fed males and females with ghrelin increased 1h chow intake **(B)**. Blocking fasting-induced ghrelin signaling in overnight fasted rats with JMV2959 decreased 1h chow intake **(C)**. All data are presented as mean ± SEM. **p < 0.01, ***p < 0.001, ****p < 0.0001 compared with respective controls. n = 12 per treatment group in each sex.

Food intake of male and female rats was assessed following 1 hour of access to chow. Fasting, tested as a means to increase endogenous ghrelin levels, increased food intake in both males and females (**Figure 3A**; **Supplementary Table 1**). However, in contrast to what we hypothesized based on the much higher fasting ghrelin levels found in females (**Figure 2A**), females ate significantly less after a period of fasting compared to males. Supporting this, 2-way ANOVA indicated a significant sex-feeding status interaction (**Supplementary Table 1**).

Upon exogenous IP ghrelin delivery, both males and female ate more without any apparent sex differences in the amount eaten or sex and treatment interaction (**Figure 3B**; **Supplementary Table 2**).

To block fasting-induced increase in ghrelin signaling, a GHSR_1A_ antagonist, JMV2959, was applied IP. This treatment reduced fasting-induced food intake at refeeding in comparison to saline-treated rats in both sexes (**Figure 3C**). Both vehicle and JMV2959-injected males ate more than females. Two-way ANOVA did not indicate a sex by treatment interaction (**Supplementary Table 3**).

### Anxiety-Like Behavior in Response to Fasting Differs by Sex

**Figure 4:**
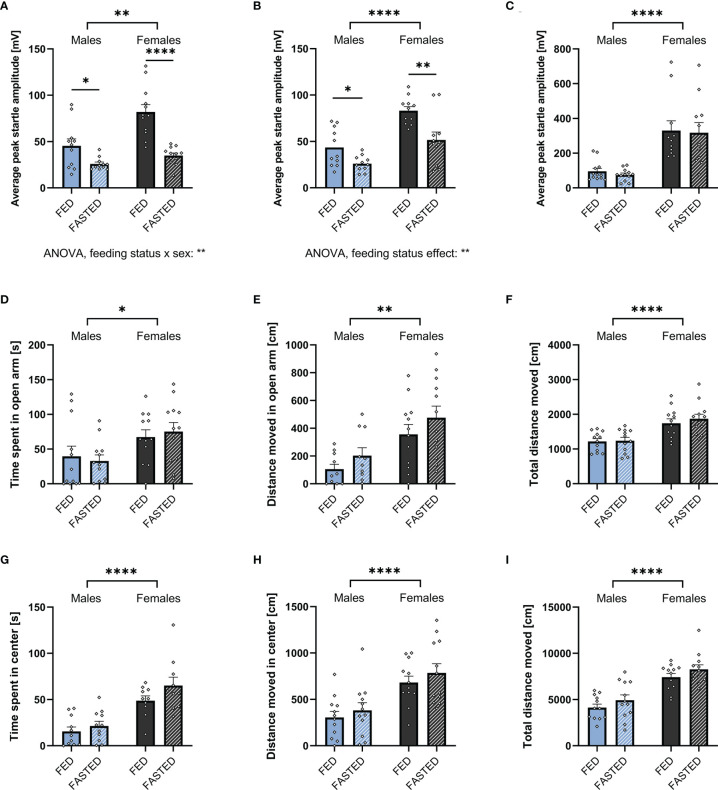
Overnight fasting is anxiolytic in the ASR test. Fasting male and female rats overnight decreased ASR amplitude following a 90 **(A)** and 95 **(B)**, but not 105 dB white noise burst **(C)**. Moreover, it did not have an effect on time spent **(D)** and distance moved **(E)** in the open arm, as well as total locomotion **(F)** in an EPM. Neither did fasting affect time spent **(G)** and distance moved **(H)** in the center, or total locomotion in the OF **(I)**. All data are presented as mean ± SEM. *p < 0.05, **p < 0.01, ****p < 0.0001 compared with respective controls. n = 12 per treatment group in each sex in the ASR and EPM, and n = 11 per treatment group in the OF respectively. ASR, acoustic startle response; EPM, elevated plus maze; OF, open field.

To assess the effect of increased endogenous ghrelin levels on anxiety-like behavior, male and female rats were either fasted overnight or had *ad libitum* access to food prior to ASR, EPM, or OF testing. Two-way ANOVA revealed that fasting significantly decreased the ASR in males and females following the 90 and 95dB stimulus. Notably, we found a feeding status x sex interaction on the ASR following the 90dB stimulus (**Figures 4A, B**; **Supplementary Table 1**), indicating that ASR response to fasting differs by sex. Interestingly, fasting was not sufficient to decrease the ASR following the 105dB stimulus in either males and females (**Figure 4C**, **Supplementary Table 1**). Compared to males, females had a stronger ASR in all feeding conditions and stimulus intensities (**Supplementary Table 1**).

Females spent more time in the open arm of the EPM, they also moved more in the open arm and overall (**Figures 4D–F**). Similarly, females spent more time in the center of the OF, moved more in the center of the OF, and moved more overall (**Figures 4G–I**). In contrast to the interaction found in the ASR, there was no significant effect of fasting on time spent and distance moved in the EPM or OF (**Figures 4D–I**; **Supplementary Table 1**).

As fasting influenced locomotor activity, we performed an ANCOVA for time spent in the open area of the testing apparatuses with total locomotion as a covariate. The covariate, total locomotion, was significantly related to the time males spent in the open areas of the EPM (F_1,21_ = 7.672, *p* = 0.011) and OF (F_1,21_ = 24.331, *p* < 0.001). After controlling for locomotion, no significant effect of feeding status on the time spent in the open areas of the EPM (F_1,21_ = 0.312, *p* = 0.582) and OF (F_1,21_ = 0.003, *p* = 0.955). Consequently, the time males spent in the open areas of EPM and OF was not influenced by an effect of fasting on locomotion.

Similar to males, an effect of total locomotion on time spent in the open areas of the EPM (F_1,21_ = 12.864, *p* = 0.002) and OF (F_1,21_ = 13.266, *p* = 0.002) has been found in females. Feeding status had no effect on the time spent in the open arms of the EPM after controlling for locomotion (F_1,21_ = 0.010, *p* = 0.920). In contrast., feeding status significantly affected the time spent in the center of the OF (F_1,21_ = 12.059, *p* = 0.002). Therefore, fasting increased the time females spent in the center of the OF after adjusting for locomotion.

### Anxiolytic Effect of the Exogenous Ghrelin Administration

**Figure 5:**
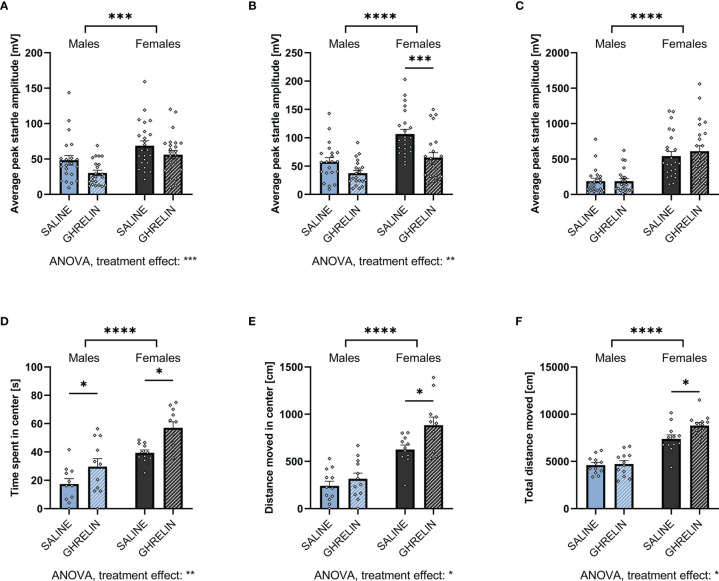
Ghrelin administration is anxiolytic in the ASR and OF. Treating *ad libitum* fed male and female rats with ghrelin did not affect the ASR following a 90dB white noise burst **(A)**. At 95dB, only ghrelin-treated females displayed a decreased startle amplitude **(B)**, however, at 105dB no significant difference was detected in both sexes **(C)**. In the OF, ghrelin treatment increased the time in the center **(D)**. Only females also increased the distance moved in the center **(E)** as well as the total locomotion in the OF when treated with ghrelin **(F)**. All data are presented as mean ± SEM. *p < 0.05, ***p < 0.001, ****p < 0.0001 compared with respective controls. n = 21 (males) and n = 23 (females) per treatment group in each sex in the ASR and n = 12 in the OF per treatment group respectively. ASR, acoustic startle response; OF, open field.

Male and female rats received IP injections with either vehicle or acyl ghrelin (0.33mg/kg) to determine whether this mode of ghrelin application affects anxiety-like behavior in both sexes and whether the behavioral responses are sex divergent. Also, here females had a higher ASR in all feeding conditions and stimulus intensities compared to males. Ghrelin administration decreased the ASR in the two lower stimulus intensities, in line with our findings during fasting. Although here the anxiolytic response was significant only in females at 95 dB (**Figures 5A, B** respectively). A significant effect of treatment was supported by two-way ANOVA (**Supplementary Table 2**). No differences in ASR were found following the 105dB stimulus (**Figure 5C**; **Supplementary Table 2**).

An anxiolytic effect was also indicated by the OF test, where ghrelin treatment significantly increased time spent in the center in both males and females (**Figure 5D**). However, the distance moved in the center and total locomotion were significantly increased only in females (**Figures 5E, F**). Here, two-way ANOVA revealed a significant effect of treatment in time spent and distance moved in the center, as well as total locomotion in the OF. For the latter, a trend for treatment x sex interaction was detected as well (**Supplementary Table 2**).

Here, ANCOVA revealed no significant effect of locomotion on time males spent in the center of the OF (F_1,21_ = 1.831, *p* = 0.192). Controlling for locomotion revealed no effect of ghrelin treatment on center time (F_1,21_ = 2.831, *p* = 0.110). In females, no significant effect of locomotion on center time was detected (F_1,21_ = 1.999, *p* = 0.173). After controlling for locomotion, a significant effect of ghrelin treatment on center time was revealed (F_1,21_ = 7.989, *p* = 0.011). Therefore, similar to fasting, ghrelin treatment increased the time females spent in the center of the OF after adjusting for locomotion.

### Blockade of Fasting-Induced Ghrelin Signaling Is Anxiogenic

To assess the role of endogenous ghrelin on anxiety-like behavior, overnight fasted rats were injected IP with the ghrelin antagonist JMV2959 (3mg/kg) or vehicle.

**Figure 6:**
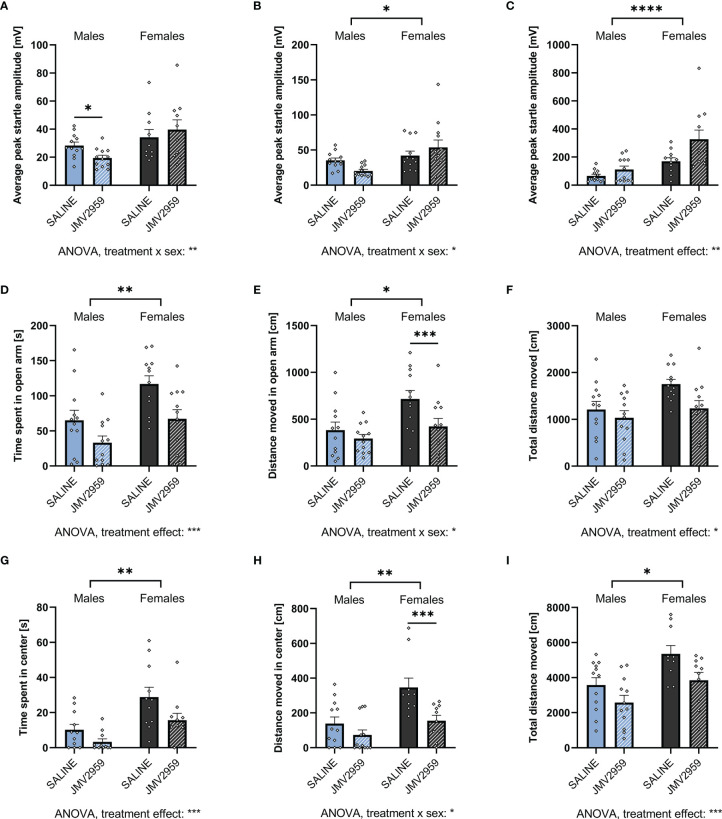
Blocking endogenous ghrelin signaling is anxiogenic in ASR, EPM, and OF. Treating overnight fasted male and female rats with the ghrelin antagonist JMV2959 decreased the ASR after a 90dB white noise burst in males only **(A)**. At 95dB, no significant differences were detected in both males and females **(B)**. However, when exposed to a 105dB white noise burst, only JMV2959-treated females displayed an elevated startle response **(C)**. In the EPM, both males and females decreased the time spent in the open arm **(D)**, but only females also decreased the locomotion in the open arm **(E)**, and overall **(F)**. When tested in the OF, a decrease in time spent **(G)** and distance moved **(H)** in the center was only detected in females. Overall locomotion, however, was reduced in both sexes **(I)**. All data are presented as mean ± SEM. *p < 0.05, **p < 0.01, ***p < 0.01, ****p < 0.0001 compared with respective controls. n = 12 per treatment group in each sex in the ASR and EPM, and n = 11 per treatment group in the open field respectively. ASR, acoustic startle response; EPM, elevated plus maze; OF, open field.

Ghrelin receptor antagonist application induced a sex divergent response in the ASR test. In females, a trend for an anxiogenic response was identified with an increase in ASR amplitude at 90 and 95 dB (**Figures 6A, B** respectively), and a pronounced increase after the 105dB stimulus (**Figure 6C**). In males on the other hand, treatment with the ghrelin antagonist surprisingly reduced anxiety-like behavior as shown by reduced ASR at the 90dB stimulus (**Figure 6A**). However, at 105dB (**Figure 6C**), the response was trending in the opposite direction and in line with the increased anxiety-like behavior measured in females, as well as in line with the hypothesis that blockade of ghrelin receptor is anxiogenic. Two-way ANOVA was able to detect a strong treatment x sex interaction at the two lower stimulus intensities, as well as a significant overall effect of treatment after the 105dB stimulus (**Supplementary Table 3**).

When assessed by EPM, treatment with JMV2959 robustly decreased time spent in the open arms in both males and females (**Figure 6D**), but only decreased distance moved in the open arm in females (**Figure 6E**). A strong reduction of overall locomotion was detected in female rats, whereas in males only a trend was discovered (**Figure 6F**). Similarly, when tested by OF, JMV2959 treatment decreased time spent in the center in both sexes (**Figure 6G**) and distance moved only in females (**Figure 6H**), as well as overall locomotion (**Figure 6I**) in both sexes. A significant overall effect of treatment on all parameters evaluated from the EPM was detected by two-way ANOVA. Additionally, we found an effect of treatment x sex interaction on the distance moved in the open arm as well as overall in the EPM, and the center of the OF (**Supplementary Table 2**). When analyzed by ANCOVA, we detected an effect of total locomotion on the time spent in the open areas of the EPM (F_1,21_ = 30.989, *p* < 0.001) and OF (F_1,21_ = 13.571, *p* = 0.002) in males. Controlling for locomotion revealed a trend for an effect of treatment on time spent in the open areas of the EPM (F_1,21_ = 3.668, *p* = 0.069), but not OF (F_1,21_ = 1.523, *p* = 0.232). In females, we found an effect of total locomotion on the time spent in the open areas in both EPM (F_1,21_ = 30.050, p < 0.001) and OF (F_1,21_ = 13.266, *p* = 0.002). However, no effect of treatment on the time spent in the open areas of the EPM (F_1,21_ = 1.127, *p* = 0.300) and OF (F_1,21_ = 0.852, *p* = 0.369) was revealed by controlling for locomotion. Accordingly, blockade of endogenous ghrelin signaling reduced time spent in the open area of the apparatuses mainly by reducing overall locomotion.

## Discussion

The orexigenic stomach-produced hormone, ghrelin, well-established for its role in feeding behavior control, has been recently indicated as a mediator of stress-related and anxiety disorders (12, 13), and is explored as a potential therapeutic target. The prevalence of anxiety disorders in humans differs by sex (27, 28), yet, sex differences in ghrelin axis and how any potential differences may affect the functional outcome of ghrelin for example on feeding or especially anxiety remain largely unexplored. Here, we evaluated key molecular and behavioral aspects of the ghrelin system in male and female rats, with a particular focus on ghrelin’s role in feeding and anxiety-like behavior.

### Sex Differences in Key Ghrelin Axis Molecules in the Fed State

Female rats displayed remarkably high levels of acylated ghrelin in blood serum compared to males. Higher plasma ghrelin levels in females found here are in line with some (29), but not all previous studies in rats. However, they align well with the difference found in adolescent (30) and adult humans (31). Why the amount of circulating ghrelin differs so drastically and whether the effect on downstream targets differs remains unclear. Ghrelin axis effect was further amplified in females by lower hepatic expression of the endogenous ghrelin receptor antagonist LEAP-2 in females. LEAP-2 operates in a non-competitive manner and reduces the ghrelin-induced activation of GHSR_1A_ (11). Serum levels of LEAP-2, in contrast to ghrelin, typically decrease during fasting and rise after feeding (4, 10, 11) and heavily depend on hepatic LEAP-2 expression (32). It is possible that the higher levels of ghrelin observed here contributed directly to the reduced LEAP-2 expression since ghrelin was previously shown to suppress LEAP-2 expression *via* AMP kinase activation (32).

The functional output of the ghrelin axis is not only dependent on the circulating active ghrelin and LEAP-2, but also the levels of available ghrelin receptor, GHSR_1A._ This receptor is widely expressed throughout the central nervous system and specifically concentrated in brain areas underlying feeding and emotionality control including the hypothalamus, hippocampus and amygdala (14, 15). While GHSR_1A_ expression has been widely investigated from rodents to humans, potential sex differences in expression patterns received a lot less attention. Here we found that females express more GHSR_1A_ in brain areas relevant to anxiety and feeding, namely the hippocampus, amygdala, and the LH. This increased receptor expression could indicate a higher sensitivity to the ghrelin signal in these brain areas, and thus a stronger behavioral response. These data are also in line with our previous study which demonstrated a higher GHSR_1A_ expression in the LH, blockade of which resulted in a reduction of food intake and motivated behavior in female but not male rats (33). However, other than in the LH, GHSR_1A_ expression did not differ between sexes in other brain areas associated with feeding behavior control: the ARC, and the DVC, or the PVN.

### Fasting Potentiates Sex Differences in Plasma Ghrelin

Fasting predictably increased plasma ghrelin levels in both males and females. However, the baseline sex differences in plasma ghrelin were potentiated further. The ghrelin levels detected in fasted males were still only 50% of the levels found in fed females, and nearly 20% of the ghrelin levels detected in fasted females. In contrast, LEAP-2 expression was only significantly decreased in fasted males. Although, as the LEAP-2 expression was already very low in females, it is likely we reached a basement effect. The ghrelin axis sex differences in a fasting state were not due to a differential metabolic response to fasting, as fasting affected blood glucose levels to a similar extent in both sexes.

Sex divergence was also detected at the level of GHSR_1A_ expression in the brain after fasting. Fasting increased GHSR_1A_ mRNA expression in the amygdala compared to *ad libitum* fed controls only in females. This is somewhat surprising as females already had a much higher GHSR_1A_ expression in this brain areas in the fed state. If increased GHSR_1A_ reflects elevated ghrelin signaling, differential impact on anxiety-like behavior in males and females after fasting is plausible. In the ARC GHSR_1A_ expression was not significantly altered in either sex, however there was a strong trend for an increased expression in males. Interestingly, we did not detect a previously indicated increase in ghrelin receptor expression in the DVC (34). However, in contrast to Huang and colleagues, who fasted the rats for at least 24h, we applied only overnight fasting. Thus, it is possible that increased GHSR_1A_ expression reported in the DVC is a response to starvation rather than moderate negative energy balance.

### High Ghrelin Levels in Females Are Driven by Ovarian Hormones

Gonadal hormones, specifically estradiol, modulate female ingestive behavior (21, 25). Based on the drastic plasma ghrelin level differences in males and females, we hypothesized that gonadal hormones contribute to this difference. To this end we evaluated serum ghrelin levels of gonadectomized male and female rats. We found that lack of gonadal steroids in ORX males did not have any effect on ghrelin levels, whereas serum ghrelin levels in OVX females were drastically reduced and indistinguishable from ghrelin levels found in males. While a previous study indicated a similar effect of gonadectomy (35), also the opposite has been reported (21, 36). Matsubara et al. (37) found a transient (present 3 or 5d after surgery) increase in ghrelin in 4wk old rats (pre-pubertal). In 9wk old (adult) rats they did not find significant increase in ghrelin *via* northern blot and a small increase in peptide was suggested by IHC 3 days post-surgery, but not 1 day, 5 days or 7 days post-surgery. Given that all time points are within a 1-7 days of OVX, it is likely that a substantial amount of ovarian hormones still remained in the circulation. Here we chose a much later time point (3 months) post OVX for ghrelin measurements. It is also likely that in the Matsubara et al. study, the small transient increase in ghrelin 3 days post-surgery is due to rebound weight gain post-surgery. Rats typically lose weight for 24-48h post any surgical intervention and on days 3-5 post surgery tend to make up the weight lost by increased feeding, thus a transient increase in ghrelin to support this transient hyperphagia is a strong possibility.

Removal of ovaries results in an array of hormonal changes and does not allow to distinguish the consequences of loss of estradiol from loss of progesterone. Therefore, to elucidate whether the drop in ghrelin levels after OVX we detected here are specifically driven by loss of estradiol, we asked whether estradiol replacement in OVX females was sufficient to rescue the reduced levels of plasma ghrelin. Here we replicated our earlier finding, and found a robust decrease in ghrelin in OVX females with vehicle treatment. Importantly, estradiol replacement partially restored ghrelin levels, indicating that estradiol plays a role in ghrelin synthesis or maintenance of circulating ghrelin levels. Role of estradiol in GHSR_1A_ expression has been previously suggested in rats (38) and humans (39), together with our current data, this indicates that estradiol is capable of controlling different levels of ghrelin axis. Further, while this study focused on differences between the sexes, future studies are needed to investigate the impact of cyclic variation of gonadal steroids in females on ghrelin signal production and reception.

Body weight could be a potentially confounding factor in assessment of circulating ghrelin levels, since OVX rats with vehicle treatment, and no estradiol replacement, were significantly heavier than intact females at the time of plasma collection, and obesity is associated with reduced ghrelin levels (19). However, estradiol replacement restored body weight of OVX rats, while ghrelin levels were still reduced compared to intact females, indicating that body weight alone is not responsible for the differences. In addition, a regression analysis of ghrelin levels and body weight also indicated no association between body weight and ghrelin levels in OVX or estrogen replaced OVX rats. Only heavier intact female rats tended to have lower levels of ghrelin in line with previous literature. It is also possible that mammalian ovaries represent an additional source of ghrelin in females (40–43). Therefore, depending on the extent of their contribution to circulating ghrelin levels, OVX may have a direct contribution to the reduced circulating ghrelin levels in addition to the indirect estradiol-mediated effect on stomach-produced ghrelin levels. This is potentially in line with the fact that estradiol did not fully restore circulating ghrelin levels. Although, to date the ovarian ghrelin fluctuations were not detected in the circulation, making it less likely that removal of the ovary would contribute to lower circulating ghrelin levels (37).

To further determine whether estradiol drives increased ghrelin levels by increasing ghrelin synthesis in the stomach, ghrelin mRNA expression in the stomach was measured in intact, OVX and OVX females with estradiol replacement. In line with this idea, we observed two-fold higher ghrelin expression in the stomach of OVX females with estradiol replacement compared to OVX females without estradiol replacement. Surprisingly though, the estradiol replacement group also had higher *ghrl* expression compared to intact females. Thus, while our data clearly support an effect of estradiol on *ghrl* expression, they do not explain all the differences we found in circulating acyl-ghrelin, at least not the higher levels of plasma ghrelin in intact females compared to those with estradiol replacement. It is plausible that posttranslational enzymatic ghrelin processing, like that executed by the enzyme GOAT (44, 45), counteracts the very high *ghrl* expression levels found in OVX estradiol replaced females. Besides, studies suggest that mammalian ovaries can be a source of ghrelin, too (40–43). Thus, measuring levels of GOAT in the stomach remains an interesting future direction for further exploring sex differences in the ghrelin axis.

### Fasting and Ghrelin Modulate Anxiety-Like Behavior in a Stimulus- and Sex-Dependent Manner

Although an association between negative energy balance and anxiety-like behavior has been shown by several previous studies, conflicting direction of effect has been reported by these studies. For instance, IP and intra-amygdala administration in male Wistar rats increase freezing in the Pavlovian fear conditioning paradigm (46). In this case, food was freely available and food availability appears to play a key role in ghrelin’s effect on anxiety. We have previously found that intra-amygdala ghrelin administration in male rats is anxiolytic in the EPM, but only when food is not available after injection (47). Studies investigating sex differences in ghrelin-modulated anxiety-like behavior under the same conditions are scarce, therefore our study sought to test both males and females under the same feeding conditions, with no food available after treatment and during testing, in complementary paradigms of anxiety-like behavior.

Overnight fasting and also ghrelin administration to *ad libitum* fed rats were anxiolytic as indicated by the ASR test. Both treatments decreased the ASR in the two lower stimulus intensities for both males and females, independent of sex differences in baseline startle amplitude. The anxiolytic effect was stronger in females than in males. These results are in line with previous studies showing that food restriction reduces ASR (24, 48). Here, however, at the highest stimulus intensity (105 dB), there was no effect of fasting or ghrelin treatment, potentially indicating ghrelin-signaling is sufficient to lower anxiety-like behavior only in moderately anxiogenic conditions. As the ASR is a defensive behavior based on a rapid skeletal muscle contraction (49), this seems reasonable when taking an evolutionary perspective: in a state of negative energy balance, anxiety-like behavior should be reduced just enough to facilitate food seeking behavior and risk potential exposure to a predator. Nonetheless, if the environment appears too dangerous (in this case represented by a 105dB white noise burst), flight responses should still be triggered irrespective of hunger.

In the current study, males had a lower baseline ASR compared to females across all experiments, i.e. males were less anxious based on ASR. Thus, the small effect size of overnight fasting or ghrelin treatment in males could be due to a basement effect. Yet, at the 105dB stimulus no effect was detected either, even though the ASR was higher compared to the response to the lower stimulus intensities.

Ghrelin’s anxiolytic effect suggested by the ASR test was further confirmed by the OF test, as exogenous ghrelin applied to *ad libitum* fed rats was sufficient to significantly decrease anxiety-like behavior displayed by both sexes in the OF. Intriguingly, only females travelled a greater distance in the center or overall in response to ghrelin. This is somewhat surprising as females already move three-fold more than males at baseline.

Overnight fasting did not have a statistically significant effect on anxiety-like behavior in the EPM and OF when interpreted by raw data of time spent and distance moved in the open areas alone. Importantly, however, in females, but not males, statistical removal of the locomotor component revealed an anxiolytic effect of fasting, represented by an increased time spent in the center of the OF. Thus, it appears that females do show anxiolysis in response to fasting also in the OF test, but locomotor activity necessary for this test confounds the interpretation of the test if locomotion is not considered when interpreting the data.

Fasting or exogenous ghrelin application experiments allow us to answer questions on the sufficiency of physiologically or pharmacologically increased ghrelin levels to alter behavior, but they do not attend to the necessity of the ghrelin system in behavioral control. This question was answered here by applying a GHSR_1A_ antagonist, JMV2959 to overnight fasted rats, thus rats with physiologically high levels of ghrelin. Ghrelin receptor antagonist application revealed a sex divergent response in the ASR test. In line with our hypothesis, JMV2959-injected female rats displayed increased anxiety-like behavior in the ASR, EPM, and OF. Ghrelin receptor antagonist was also associated with increased anxiety-like behavior in male rats, but only in the OF and EPM tests. Importantly, for both sexes statistically accounting for locomotor activity in the OF and EPM tests revealed that the anxiety scores are largely driven by the overall lower locomotor activity induced by JMV2959. Thus, taken together a clear anxiogenic effect of GHSR_1A_ blockade was detected only in females. Counterintuitively, males treated with ghrelin antagonist displayed a decrease in startle amplitude at the lower stimulus intensities, and no significant effect after the strongest noise burst.

### Integrating Molecular and Behavioral Sex Differences Found

The fasting-induced upregulation of GHSR_1A_ mRNA in the female amygdala, and the already higher expression at baseline, may be in line with a higher dependence of female rats on ghrelin signal to control anxiety-like behavior. Ghrelin crosses the blood brain barrier *via* transport and passive diffusion (50–53). Therefore, ghrelin’s influence on female anxiety-like behavior could be a result of the increased circulating ghrelin directly binding to amygdala ghrelin receptors. Alternatively, upregulation of the ghrelin receptor itself might induce an anxiolytic effect. The ghrelin receptor exerts a high constitutive activity, which alone is able to increase intracellular calcium levels (54). Viral overexpression of the GHSR_1A_ in the amygdala of mice is anxiolytic (2). As females expressed higher levels of GHSR_1A_ mRNA in the hippocampus compared to males, it is possible that the hippocampus indirectly relays ghrelin signaling to the amygdala. In males, fasting tended to increase ghrelin receptor mRNA in the ARC. Traditionally, ghrelin signaling in the ARC is associated with the induction of feeding behavior (55). However, ghrelin administration IP (46) and into the ARC was anxiogenic in male rats (56), therefore antagonizing fasting-induced ghrelin signaling could potentially have the opposite effect. Since we applied the antagonist systemically, all GHSR_1A_ populations are potentially accessible, thus it is plausible that fasting in females increases the ghrelin signal reception in amygdala and in males in the ARC, leading to the sex divergent effects of receptor blockade on behavior. In line with the differential GHSR_1A_ receptor expression after fasting, fasting had an opposite sex divergence on feeding behavior, compared to anxiety-like behavior. Fasting and ghrelin administration significantly increased food intake in both sexes which is consistent with previously reported data (21), however this effect was stronger in males compared to females.

Combined with the molecular sex differences we found, namely the higher circulating ghrelin levels, reduced LEAP-2, and increased ghrelin receptor expression in emotionality-influencing brain areas of females, the sex divergent response to ghrelin signaling in feeding and anxiety-like behavior suggests that the male response to negative energy balance is primed to increase food intake, whereas the female response largely affects anxiety-like behavior besides food intake. This is likely needed as females show more anxiety-like behavior at baseline. One evolution-based explanation for this divergence is that food scarcity could pose a bigger complication for females as they often care for offspring, and stronger anxiolysis could better ensure the survival of the female and progeny. Similarly, if there is no need for food, the anxiogenic effect is stronger, considering the higher risk to females and by extension offspring, if unnecessary risks are taken. In line with the idea, ghrelin levels are more prominently affected by chronic early-life stress in neonatal female mice compared to males (57).

Metabolic and psychiatric disorders are major public health concerns; many studies support associations between the two. It is likely that the same mechanisms underlie these different classes of disorders. Changes in gut-brain communication, like those conveyed by ghrelin, represent one such mechanism. Here, we show that female rats appear to be wired for higher sensitivity to fasting-induced anxiolytic ghrelin signaling. Further, the sex difference we found in the ghrelin axis is modulated, at least partly, at the level of synthesis, by gonadal steroids, specifically estradiol. Apart from the modulation of ingestive behavior, ghrelin plays a more prominent role in the regulation of anxiety-like behavior of female rats. Therefore, when exploring ghrelin as a target for the treatment of e.g. eating, affective or stress disorders, our data suggest that it is crucial to account for sex, and expend the range of behaviors tested to accurately determine the overall impact of altering ghrelin axis signaling.

## Data Availability Statement

The original contributions presented in the study are included in the article/**Supplementary Material**. Further inquiries can be directed to the corresponding author.

## Ethics Statement

All animal procedures were carried out with ethical permission from the Animal Welfare Committee of the University of Gothenburg and Jordbruksverket, in accordance with legal requirements of the European Community (Decree 35-970/96, 137/15, and 1-2019).

## Author Contributions

SB, KS, and J-PK contributed to conception and design of the study. SB, IM, and MAs performed the surgeries. SB and MAb carried out the experiments. SB, J-PK, JC, MAb, FL, MAs, and JER contributed to sample collection. SB and KS processed the experimental data, performed the analysis, drafted the manuscript, and designed the figures. All authors contributed to manuscript revision, read, and approved the submitted version.

## Funding

This research was funded by the Wallenberg Foundation (WCMTM), Swedish Research Council (2018-00660 to KS), Ragner Söderberg Foundation (KS), and the Swiss National Science Foundation (183899 to J-PK).

## Conflict of Interest

The authors declare that the research was conducted in the absence of any commercial or financial relationships that could be construed as a potential conflict of interest.

## Publisher’s Note

All claims expressed in this article are solely those of the authors and do not necessarily represent those of their affiliated organizations, or those of the publisher, the editors and the reviewers. Any product that may be evaluated in this article, or claim that may be made by its manufacturer, is not guaranteed or endorsed by the publisher.
