# Supplementary material for: From an Empty Stomach to Anxiolysis: Molecular and Behavioral Assessment of Sex Differences in the Ghrelin Axis of Rats
Source: Front Endocrinol (Lausanne). 2022 Jun 16;13:901669. doi: 10.3389/fendo.2022.901669 (PMC9243305; doi:10.3389/fendo.2022.901669)
Supplement: Supplementary file 1 [file DataSheet_1.docx]

Supplementary Material

# Supplementary Figures

**
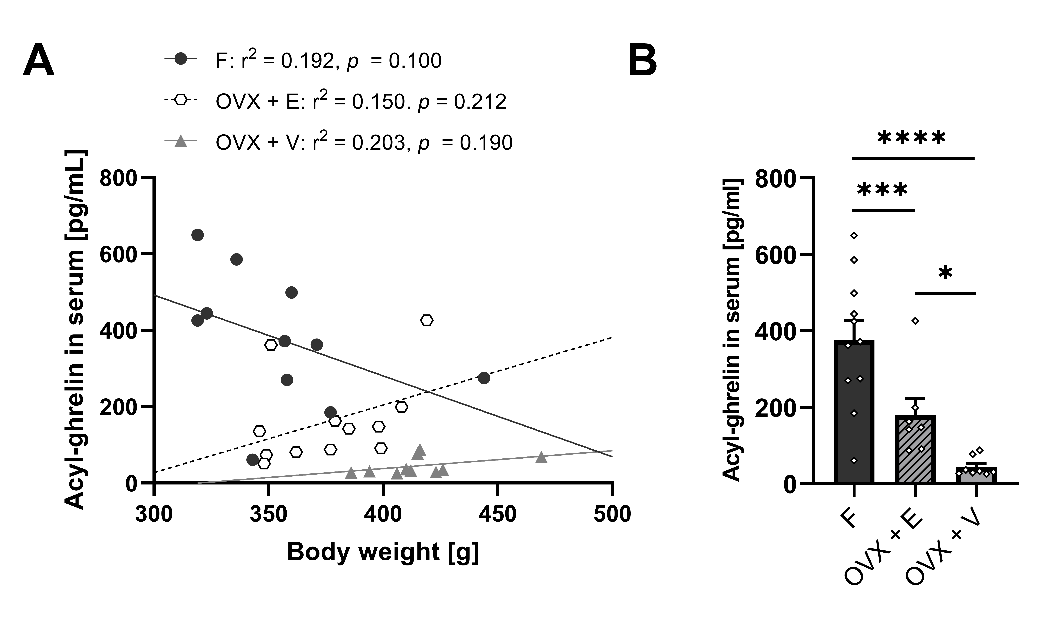
**

**Supplementary Figure 1.** Linear regression of serum acyl-ghrelin levels of intact females (n = 11), OVX females with (OVX + E, n = 12), and without estradiol replacement (OVX + V, n = 10) with body weight as the dependent variable (A). Comparison of acyl-ghrelin levels of intact (n = 11), OVX + E (n = 7), and OVX + V (n = 8), in similar bodyweight group (400 ± 20g) (B). All data are presented as mean ± SEM. * *p* < 0.05, *** *p* < 0.001, **** *p* < 0.0001 compared with respective controls.

# Supplementary Tables

**Supplementary Table 1.** Results of two-way ANOVAs comparing key parameters of the behavior of fed and fasted male and female rats in the ASR, EPM and OF test. Factors: feeding status (fasted vs. fed) and sex (male vs. female).

| **Location of graphed data** | **Parameters measured** | **Two-way ANOVA results** | | |
| --- | --- | --- | --- | --- |
|  |  | Variation source | F (DFn, DFd) | *p* value |
| Fig 2B | Acyl-Ghrelin levels in serum [pg/ml] | Feeding status x Sex | F (1, 36) = 26,95 | P<0,0001 |
|  |  | Feeding status | F (1, 36) = 76,70 | P<0,0001 |
|  |  | Sex | F (1, 36) = 324,9 | P<0,0001 |
| Fig 2A | Blood glucose [mmol/L] | Feeding status x Sex | F (1, 44) = 1,097 | P=0,3007 |
|  |  | Feeding status | F (1, 44) = 114,8 | **P<0,0001** |
|  |  | Sex | F (1, 44) = 0,1622 | P=0,6891 |
| Fig 2C | LEAP-2 expression [relative to GAPDH] | Feeding status x Sex | F (1, 44) = 0,2484 | P=0,6236 |
|  |  | Feeding status | F (1, 44) = 30,06 | P<0,0001 |
|  |  | Sex | F (1, 44) = 0,06211 | P=0,8057 |
| Fig 3A | 1h Chow intake [g]  *Within subject* | Feeding status x Sex | F (1, 22) = 20,98 | P=0,0001 |
|  |  | Feeding status | F (1, 22) = 217,8 | P<0,0001 |
|  |  | Sex | F (1, 22) = 13,07 | P=0,0015 |
| Fig 4A | Average peak startle amplitude [mv] after 90 dB stimulus  *Within subject* | Feeding status x Sex | F (1, 21) = 8,120 | P=0,0096 |
|  |  | Feeding status | F (1, 21) = 47,55 | P<0,0001 |
|  |  | Sex | F (1, 21) = 12,25 | P=0,0021 |
| Fig 4B | Average peak startle amplitude [mv] after 95 dB stimulus  *Within subject* | Feeding status x Sex | F (1, 20) = 0,6331 | P=0,2256 |
|  |  | Feeding status | F (1, 20) = 10,71 | P=0,0003 |
|  |  | Sex | F (1, 20) = 24,79 | P<0,0001 |
| Fig 4C | Average peak startle amplitude [mv] after 105 dB stimulus  *Within subject* | Feeding status x Sex | F (1, 21) = 0,006332 | P=0,9373 |
|  |  | Feeding status | F (1, 21) = 0,1441 | P=0,7081 |
|  |  | Sex | F (1, 21) = 44,14 | P<0,0001 |
| Fig 4D | Time spent in open arm of EPM [s]  *Within subject* | Feeding status x Sex | F (1, 22) = 0,4626 | P=0,5035 |
|  |  | Feeding status | F (1, 22) = 0,004525 | P=0,9470 |
|  |  | Sex | F (1, 22) = 7,347 | P=0,0128 |
| Fig 4E | Distance moved in open arm of EPM [cm]  *Within subject* | Feeding status x Sex | F (1, 22) = 0,03044 | P=0,8633 |
|  |  | Feeding status | F (1, 22) = 2,968 | P=0,1003 |
|  |  | Sex | F (1, 22) = 13,66 | P=0,0014 |
| Fig 4F | Total distance moved in EPM [cm]  *Within subject* | Feeding status x Sex | F (1, 22) = 0,1887 | P=0,6682 |
|  |  | Feeding status | F (1, 22) = 0,3417 | P=0,5648 |
|  |  | Sex | F (1, 22) = 36,53 | P<0,0001 |
| Fig 4G | Time spent in center of OF [s]  *Within subject* | Feeding status x Sex | F (1, 22) = 0,8029 | P=0,3814 |
|  |  | Feeding status | F (1, 22) = 3,615 | P=0,0725 |
|  |  | Sex | F (1, 22) = 35,33 | P<0,0001 |
| Fig 4H | Distance moved in center of OF [cm]  *Within subject* | Feeding status x Sex | F (1, 22) = 0,02509 | P=0,8756 |
|  |  | Feeding status | F (1, 22) = 1,091 | P=0,3076 |
|  |  | Sex | F (1, 22) = 29,73 | P<0,0001 |
| Fig 4I | Total distance moved in OF [cm]  *Within subject* | Feeding status x Sex | F (1, 22) = 0,002371 | P=0,9616 |
|  |  | Feeding status | F (1, 22) = 2,213 | P=0,1511 |
|  |  | Sex | F (1, 22) = 82,00 | P<0,0001 |

**Supplementary Table 2.** Results of two-way ANOVAs comparing key parameters of the behavior of saline and ghrelin treated male and female rats in the ASR, EPM and OF test. Factors: treatment (saline vs. ghrelin) and sex (male vs. female).

| **Location of graphed data** | **Parameters measured** | **Two-way ANOVA results** | | |
| --- | --- | --- | --- | --- |
|  |  | Variation source | F (DFn, DFd) | *p* value |
| Fig 3B | 1h Chow intake [g]  *Within subject* | Treatment x Sex | F (1, 22) = 2,873 | P=0,1042 |
|  |  | Treatment | F (1, 22) = 77,41 | P<0,0001 |
|  |  | Sex | F (1, 22) = 2,772 | P=0,1101 |
| Fig 5A | Average peak startle amplitude [mv] after 90 dB stimulus  *Within subject* | Treatment x Sex | F (1, 44) = 0,2084 | P=0,6503 |
|  |  | Treatment | F (1, 44) = 7,423 | P=0,0092 |
|  |  | Sex | F (1, 44) = 13,63 | P=0,0006 |
| Fig 5B | Average peak startle amplitude [mv] after 95 dB stimulus  *Within subject* | Treatment x Sex | F (1, 44) = 2,048 | P=0,1598 |
|  |  | Treatment | F (1, 42) = 17,03 | P=0,0002 |
|  |  | Sex | F (1, 42) = 25,56 | P<0,0001 |
| Fig 5C | Average peak startle amplitude [mv] after 105 dB stimulus  *Within subject* | Treatment x Sex | F (1, 44) = 0,4417 | P=0,5098 |
|  |  | Treatment | F (1, 44) = 0,4381 | P=0,5115 |
|  |  | Sex | F (1, 44) = 35,97 | P<0,0001 |
| Fig 5D | Time spent in center of OF [s]  *Within subject* | Treatment x Sex | F (1, 22) = 0,4516 | P=0,5093 |
|  |  | Treatment | F (1, 22) = 13,57 | P=0,0015 |
|  |  | Sex | F (1, 22) = 34,66 | P<0,0001 |
| Fig 5E | Distance moved in center of OF [cm]  *Within subject* | Treatment x Sex | F (1, 22) = 2,113 | P=0,1608 |
|  |  | Treatment | F (1, 22) = 6,888 | P=0,0158 |
|  |  | Sex | F (1, 22) = 64,15 | P<0,0001 |
| Fig 5F | Total distance moved in OF [cm]  *Within subject* | Treatment x Sex | F (1, 22) = 3,154 | P=0,0896 |
|  |  | Treatment | F (1, 22) = 4,331 | P=0,0493 |
|  |  | Sex | F (1, 22) = 101,9 | P<0,0001 |

**Supplementary Table 3.** Results of two-way ANOVAs comparing key parameters of the behavior of saline and JMV2959 treated fasted male and female rats in the ASR, EPM and OF test. Factors treatment (saline vs. JMV2959) and sex (male vs. female).

| **Location of graphed data** | **Parameters measured** | **Two-way ANOVA results** | | |
| --- | --- | --- | --- | --- |
|  |  | Variation source | F (DFn, DFd) | *p* value |
| Fig 3C | 1h Chow intake [g]  *Within subject* | Feeding status x Sex | F (1, 22) = 0,2697 | P=0,6087 |
|  |  | Feeding status | F (1, 22) = 21,59 | P=0,0001 |
|  |  | Sex | F (1, 22) = 18,48 | P=0,0003 |
| Fig 6A | Average peak startle amplitude [mv] after 90 dB stimulus  *Within subject* | Treatment x Sex | F (1, 22) = 9,328 | P=0,0063 |
|  |  | Treatment | F (1, 22) = 0,5046 | P=0,4857 |
|  |  | Sex | F (1, 22) = 5,154 | P=0,0344 |
| Fig 6B | Average peak startle amplitude [mv] after 95 dB stimulus  *Within subject* | Treatment x Sex | F (1, 22) = 6,751 | P=0,0164 |
|  |  | Treatment | F (1, 22) = 0,1108 | P=0,7424 |
|  |  | Sex | F (1, 22) = 7,030 | P=0,0146 |
| Fig 6C | Average peak startle amplitude [mv] after 105 dB stimulus  *Within subject* | Treatment x Sex | F (1, 22) = 2,671 | P=0,1171 |
|  |  | Treatment | F (1, 22) = 8,855 | P=0,0072 |
|  |  | Sex | F (1, 22) = 17,75 | P=0,0004 |
| Fig 6D | Time spent in open arm of EPM [s]  *Within subject* | Treatment x Sex | F (1, 22) = 0,9582 | P=0,3383 |
|  |  | Treatment | F (1, 22) = 20,68 | P=0,0002 |
|  |  | Sex | F (1, 22) = 8,333 | P=0,0086 |
| Fig 6E | Distance moved in open arm of EPM [cm]  *Within subject* | Treatment x Sex | F (1, 22) = 4,305 | P=0,0499 |
|  |  | Treatment | F (1, 22) = 15,36 | P=0,0007 |
|  |  | Sex | F (1, 22) = 5,412 | P=0,0296 |
| Fig 6F | Total distance moved in EPM [cm]  *Within subject* | Treatment x Sex | F (1, 22) = 3,772 | P=0,0650 |
|  |  | Treatment | F (1, 22) = 15,80 | P=0,0006 |
|  |  | Sex | F (1, 22) = 3,662 | P=0,0688 |
| Fig 6E | Time spent in center of OF [s]  *Within subject* | Treatment x Sex | F (1, 20) = 1,519 | P=0,2321 |
|  |  | Treatment | F (1, 20) = 15,07 | P=0,0009 |
|  |  | Sex | F (1, 20) = 10,52 | P=0,0041 |
| Fig 6F | Distance moved in center of OF [cm]  *Within subject* | Treatment x Sex | F (1, 20) = 5,852 | P=0,0252 |
|  |  | Treatment | F (1, 20) = 24,57 | P<0,0001 |
|  |  | Sex | F (1, 20) = 9,248 | P=0,0065 |
| Fig 6G | Total distance moved in OF [cm]  *Within subject* | Treatment x Sex | F (1, 20) = 0,7087 | P=0,4098 |
|  |  | Treatment | F (1, 20) = 17,01 | P=0,0005 |
|  |  | Sex | F (1, 20) = 8,028 | P=0,0103 |
